# Food‐derived hydrophilic antioxidant ergothioneine is distributed to the brain and exerts antidepressant effect in mice

**DOI:** 10.1002/brb3.477

**Published:** 2016-04-22

**Authors:** Noritaka Nakamichi, Keigo Nakayama, Takahiro Ishimoto, Yusuke Masuo, Tomohiko Wakayama, Hirotaka Sekiguchi, Keita Sutoh, Koji Usumi, Shoichi Iseki, Yukio Kato

**Affiliations:** ^1^ Faculty of PharmacyInstitute of Medical, Pharmaceutical and Health SciencesKanazawa UniversityKanazawa920‐1192Japan; ^2^ School of MedicineInstitute of Medical, Pharmaceutical and Health SciencesKanazawa UniversityKanazawa920‐1192Japan; ^3^ Life Science Institute Co. Ltd.2‐6‐6 Nihombashi‐HoridomechoChuo‐kuTokyo103‐0012Japan; ^4^ L•S Corporation Co. Ltd.3‐10‐1 Ningyocho‐NihonbashiChuo‐kuTokyo103‐0013Japan

**Keywords:** Antidepressant effect, depression, disposition, ergothioneine, neuronal differentiation, OCTN1

## Abstract

**Background:**

Clinically used antidepressants suffer from various side effects. Therefore, we searched for a safe antidepressant with minimal side effects among food ingredients that are distributed to the brain. Here, we focused on ERGO (ergothioneine), which is a hydrophilic antioxidant and contained at high levels in edible golden oyster mushrooms. ERGO is a typical substrate of carnitine/organic cation transporter OCTN1/SLC22A4, which is expressed in the brain and neuronal stem cells, although little is known about its permeation through the BBB (blood–brain barrier) or its neurological activity.

**Methods:**

To clarify the exposure of ERGO to brain and the possible antidepressant‐like effect after oral ingestion, ERGO or GOME (golden oyster mushroom extract) which contains 1.2% (w/w) ERGO was mixed with feed and provided to mice for 2 weeks, and then ERGO concentration and antidepressant‐like effect were evaluated by LC‐MS/MS and FST (forced swimming test) or TST (tail suspension test), respectively.

**Results:**

Diet containing ERGO or GOME greatly increased the ERGO concentrations in plasma and brain, and significantly decreased the immobility time in both FST and TST. The required amount of GOME (~37 mg/day) to show the antidepressant‐like effect corresponds to at most 8 g/day in humans. In mice receiving GOME‐containing diet, doublecortin‐positive cells showed a significant increase from the basal level, suggesting promotion of neuronal differentiation.

**Conclusion:**

Thus, orally ingested ERGO is transported across the BBB into the brain, where it may promote neuronal differentiation and alleviate symptoms of depression at plausibly achieved level of daily ingestion.

## Introduction

Depression is a mental illness with serious social consequences, and indeed, about 20% of the population suffers from the mental illness at some point in their lifetime (Browne and Lucki 2013). Various antidepressant therapeutic agents, including selective serotonin reuptake inhibitors, serotonin–noradrenaline reuptake inhibitors, and tricyclic antidepressants, are currently used for the treatment of depression. However, these drugs have multiple side effects (Bet et al. 2013; Alusik et al. 2014). Therefore, it would obviously be desirable to develop antidepressant drugs with few or no side effects that can be safely used in long‐term treatment. From this point of view, a useful strategy might be to search for antidepressant activity among edible products using animal models of depression. In fact, it has been reported that several compounds included in edible products exert an antidepressant‐like effect. GBE (*Ginkgo biloba* extract) shows its antidepressant‐like effect via modulation of serotonergic and dopaminergic neurotransmission, or antioxidant enzymes in rodents (Sakakibara et al. 2006; Rojas et al. 2011). *Hypericum perforatum* extract exhibits its antidepressant‐like effect by biochemical mechanisms similar to the tricyclic antidepressants or serotonin reuptake inhibitors in rodents (Bukhari and Dar 2013).

ERGO (Ergothioneine) is naturally occurring antioxidant derived from the diet and abundantly contained in golden oyster mushroom (*Pleurotus cornucopiae var. citrinopileatus*), which is an edible mushroom belonging to the family Pleurotaceae. It is hydrophilic and membrane impermeable, but is well absorbed from the gastrointestinal tract due to the presence of a specific carrier‐mediated transport system, carnitine/organic cation transporter OCTN1/SLC22A4 (Gründemann et al. 2005; Kato et al. 2010). OCTN1 is expressed ubiquitously in humans, including small intestine (Sugiura et al. 2010), neuronal bodies, and neurites (Lamhonwah et al. 2008). ERGO is taken up into neurons by OCTN1 in a Na^+^‐dependent manner (Nakamichi et al. 2012). ERGO serves as an antioxidant, scavenging hydrogen peroxide, and peroxynitrite (Aruoma et al. 1999). It prevents oxidative damage to DNA and protein (Zhu et al. 2011), and protects neurons against *β*‐amyloid‐induced cytotoxicity (Yang et al. 2012). On the other hand, in depressed patients, oxidative stress is increased and antioxidant levels are decreased (Ng et al. 2008), and interestingly, preclinical studies have suggested that antioxidants may possess antidepressant‐like effects in rodents (Eren et al. 2007; Zafir et al. 2009). In addition, ERGO exerts unique biological activities in neural cells, including promotion of neuronal differentiation (Ishimoto et al. 2014). Some other antidepressant drugs (fluoxetine, imipramine, and reboxetine) also induce neurogenesis (Malberg et al. 2000; Santarelli et al. 2003; Ohira et al. 2013), suggesting a possible association between neurogenesis and antidepressant effect. This could be supported by the finding that mice developed depression‐like symptoms, as assessed with several behavioral tests, when neurogenesis was inhibited (Snyder et al. 2011). On the other hand, neuronal atrophy was observed in the hippocampal dentate gyrus of depressed patients (Cobb et al. 2013) and might be involved in the pathophysiology of depression.

The experimental evidence mentioned above may imply possible pharmacological relevance of the biological activity of ERGO with depression. However, there is no direct evidence that ERGO has antidepressant activity. Therefore, in this study, we examined the disposition of ERGO, its antidepressant‐like effect, and the mechanism involved in mice given diet containing ERGO or GOME (golden oyster mushroom extract).

## Materials and Methods

### Materials

Powdered GOME (Aminothioneine^®^), which contains 1.2% (w/w) ERGO, and GBE were supplied by L•S Corporation (Tokyo, Japan). Mouse control feed and Basal Diet^®^ were purchased from Oriental Yeast Co. (Tokyo, Japan) and TestDiet (St. Louis, MO), respectively, and contained 0.2 *μ*g/g and less than 0.01 *μ*g ERGO/g chaw, respectively, according to our liquid chromatography‐tandem mass spectrometry (LC‐MS/MS) determination. All other chemicals and reagents were of the highest purity available and were purchased from commercial sources.

### Animals

C57BL/6J mice were purchased from Sankyo Labo Service Co. (Tokyo, Japan). About 5–8‐week‐old male mice were housed under pathogen‐free conditions at controlled temperature (21–25°C) with a 12 h light/dark cycle. The lights remained on from 8:00 to 20:00, and food and water were available ad libitum. The animals were cared for in strict compliance with the guidelines outlined in the National Institutes of Health Guide for the Care and Use of Laboratory Animals. All animal procedures used in this work were approved by the Kanazawa University Animal Care Committee.

### Measurement of ERGO

**Figure 1:**
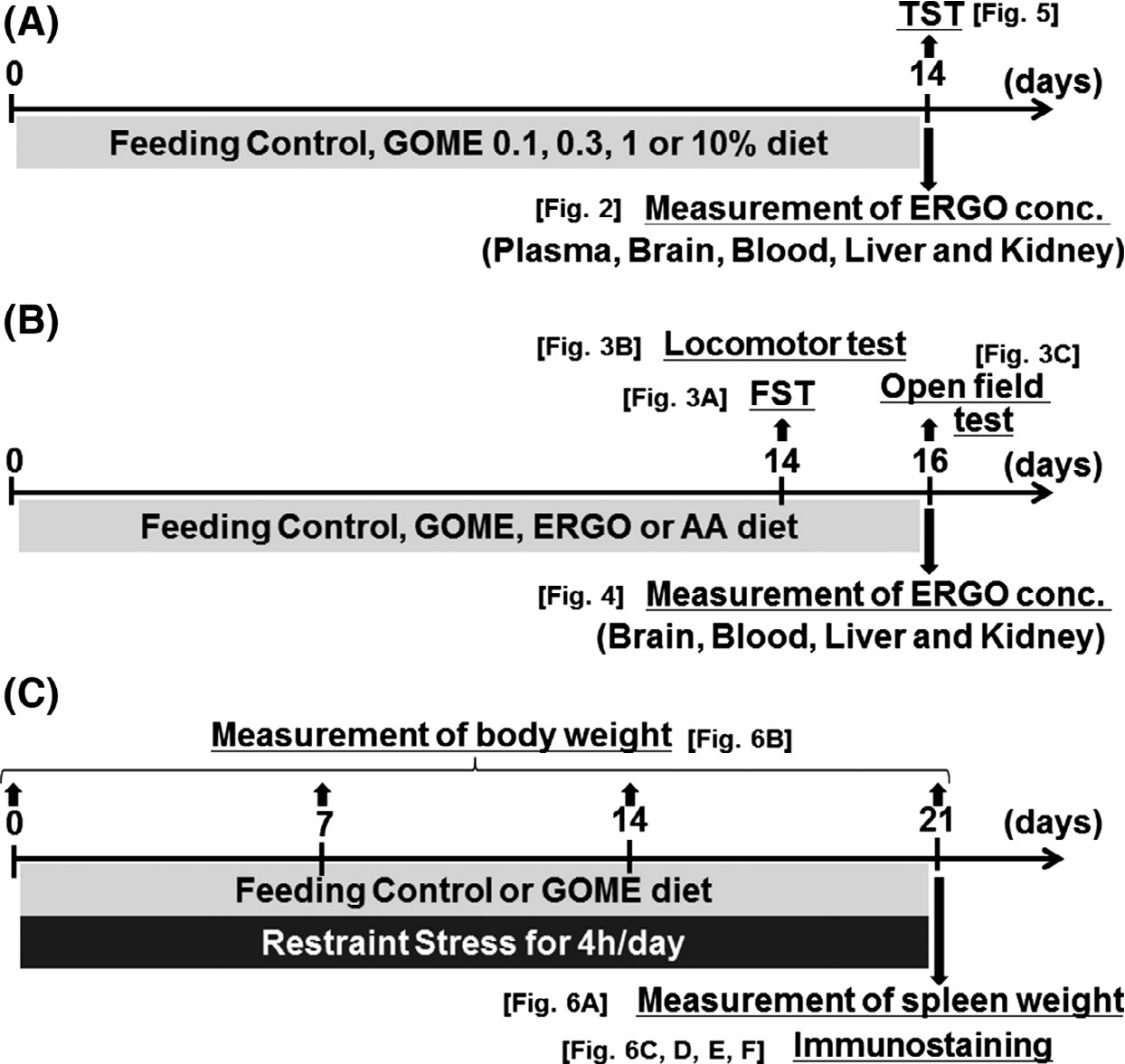
Schematic representation of experimental schedule. Each timeline shows experimental schedule for (A) Figs. 2, 5, (B) Figs. 3, 4, or (C) Fig. 6.

Mice were fed diet containing 0, 0.1, 0.3, 1, or 10% (w/w) GOME for 2 weeks, and blood was collected (Fig. 1A). The mice were then decapitated, and brain, liver, and kidney were collected. All the tissue samples were weighed, and portions were homogenized in two volumes of distilled water. The homogenates were further diluted 5‐ to 4000‐fold with water. The diluted homogenates, plasma, and blood were deproteinized with methanol and subjected to LC‐MS/MS after centrifugation. The LC‐MS/MS system was based on a model LCMS‐8040 (Shimadzu, Kyoto, Japan). Chromatography was performed by means of step‐gradient elution (flow rate, 0.3 mL/min) as follows: 0–0.5 min: B conc. 95%; 0.5–3.5 min: B conc. 95–30%; 3.5–5.5 min: B conc. 30%; 5.5–5.6 min: B conc. 30–95%; 5.6–8 min: B conc. 95% (A, water containing 0.1% formic acid; B, acetonitrile‐containing 0.1% formic acid), using a Luna 3.0 *μ*m HILIC column (200 Å, 150 × 2.0 mm; Phenomenex, Torrance, CA) at 50°C. L‐(+)‐Ergothioneine‐d9 (Toronto Research Chemicals, Toronto, Canada) was used as the internal standard. Oral clearance (CL_oral_) and tissue‐to‐plasma concentration ratio (Kp) were calculated as the ratio of the daily dose of ERGO to plasma ERGO concentration, and the ratio of tissue‐to‐plasma ERGO concentration measured at 2 weeks after the start of the ERGO‐containing diet. To calculate the daily dose of ERGO, the average daily food intake of the mice was assumed to be 3.68 g (Kato et al. 2009).

The high‐performance liquid chromatography (HPLC) analysis was carried out according to the method of Kato et al. (2010) with minor modifications. All the tissue samples were weighed, and portions were homogenized in two volumes of distilled water. Blood and homogenates were further diluted 10–30 times with water. The diluted samples were deproteinized with acetonitrile. The analytical column was a HILIC (4.6 × 250 mm; Nacalai Tesque, Kyoto, Japan). The mobile phase was 0.01 mol/L ammonium acetate and acetonitrile in a ratio of 86: 14 (flow rate 1 mL/min, detection at 260 nm). Cefoperazone was used as the internal standard.

### Locomotor activity, forced swimming, open field, and tail suspension tests

Behavioral tests were performed according to the methods of Mouri et al. (2012) with minor modifications. Mice were fed diet including 10% (w/w) GOME, ERGO (120 mg/100 g diet), 10% (w/w) GBE or AA (ascorbic acid; 120 mg/100 g diet) for 2 weeks (Fig. 1B). Pre‐FST (forced swimming test; 5 min) was performed before ingestion of each diet. After 2 weeks, to measure locomotor activity in a novel environment, each mouse was placed in a transparent acrylic cage. After 60 min of habituation, locomotion was evaluated for 5 min using a video camera and ImageJ software (National Institutes of Health, Bethesda, MD). Each mouse was placed in a 19 cm plastic cylinder (16 cm in diameter) containing 10 cm of water, which was maintained at 25°C, and forced to swim for 5 min. The immobility time was recorded on a video camera. Two days after FST, each mouse was placed in a clear acrylic box (45 × 45 × 45 cm), which was divided into three areas (center, middle, and outer zones). The center, middle, and outer zones were divided into 4, 12, and 20 sections, respectively. The mouse was placed in a section of the outer zone and then allowed to freely explore its environment. The amount of time spent in each zone was evaluated for 5 min using a video camera and ImageJ software. After the open field test, blood was collected. Brain, liver, and kidney were collected after decapitation. ERGO in these samples was quantitated by means of HPLC.

Mice were fed diet containing 0, 0.1, 0.3, 1, or 10% GOME for 2 weeks (Fig. 1A). The mice were suspended from a string attached with plastic tape to the tail, and the tail was covered with a 50 mL centrifuge tube, cut to an appropriate size, to prevent the animals climbing their tails. The distance between the tip of the nose of each mouse and the floor was about 40 cm. The mice were suspended for 3 min, and the duration of immobility from the end of min 1 to the end of min 3 was measured using images captured on a video camera. In this procedure, the immobility time of mice treated with the tricyclic antidepressant imipramine was clearly decreased compared to that of control mice (data not shown).

### Repeated restraint stress

The restraint stress procedure was carried out according to Kim et al. (2011) with minor modifications. Mice were randomly divided into four groups: nonstressed mice given control diet (*n* = 6), nonstressed mice given 10% GOME diet (*n* = 6), stressed mice given control diet (*n* = 8), and stressed mice given 10% GOME diet (*n* = 8). Stress was imposed by forcing the animals into an immobilizer (Natsume Seisakusho, Tokyo, Japan) for 4 h (10:00–14:00) each day, for 21 consecutive days (Fig. 1C). The apparatus fitted tightly around the mice, which were essentially unable to move. Body weight was measured every week. After 3 weeks, the spleens were removed and weighed.

### Bromodeoxyuridine labeling and tissue preparation

BrdU (Bromodeoxyuridine) labeling and tissue preparation were carried out according to the methods of Ogita et al. (2005) with minor modifications. To detect new cells, BrdU (50 mg/10 mL/kg) dissolved in PBS (phosphate‐buffered saline) was intraperitoneally injected into mice after repeated restraint stress experiments. The mice were given two consecutive injections of BrdU at 24 h and 12 h before decapitation. Under deep anesthesia, the mice were intracardially perfused with phosphate buffer containing 4% paraformaldehyde. The brains were quickly removed, and postfixed with the same fixative solution at 4°C overnight. Postfixed brains were embedded in paraffin, then coronal sections of 4‐*μ*m thickness were cut with a microtome and placed on Silan‐coated slide glasses. The paraffin‐embedded brain sections were deparaffinized with xylene, rehydrated with ethanol at graded decreasing concentrations of 100–50% (vol/vol), and finally washed with PBS.

### Immunostaining

Immunostaining was carried out according to the method of Jung et al. (2013) with minor modifications. Sections were incubated in sodium citrate at 92°C for 15 min, 0.3% Tween 20 in PBS for 20 min, and 3% hydrogen peroxide in PBS for 30 min. To detect BrdU incorporation, sections were further treated with 2 mol/L HCl at 35°C for 30 min. They were blocked with 4% Block Ace, then incubated with a primary antibody against BrdU (1:200; Abcam, Cambridge, MA) or DCX (doublecortin; 1:200; Santa Cruz Biotechnology, Santa Cruz, CA) at 4°C overnight, reacted with a biotinylated secondary antibody against rat or goat IgG (1:250) for 1 h at room temperature, and then reacted with streptavidin‐biotin‐peroxidase complex for 30 min at room temperature. The peroxidase reaction was visualized by incubation of the sections in diaminobenzidine/hydrogen peroxide solution. The sections were then washed three times with PBS for 3 min, mounted on gelatin‐coated slides, dehydrated in an ascending alcohol series, and cleared in xylene. Immunopositive cells in hippocampal dentate gyrus were observed using a light microscope and counted with Hybrid cell‐counting software (KEYENCE, Osaka, Japan). To obtain the total number of immunopositive cells per dentate gyrus, the area of dentate gyrus was measured with ImageJ software.

### Statistical analysis

Data are expressed as the mean ± SEM. The statistical significance of differences was determined by means of Student's *t*‐test, one‐way or repeated measures ANOVA, followed by the appropriate post hoc tests (Tukey–Kramer or Dunnett's test), and *P *<* *0.05 was regarded as denoting a significant difference.

## Results

### Brain distribution of ERGO ingested in diet

**Figure 2:**
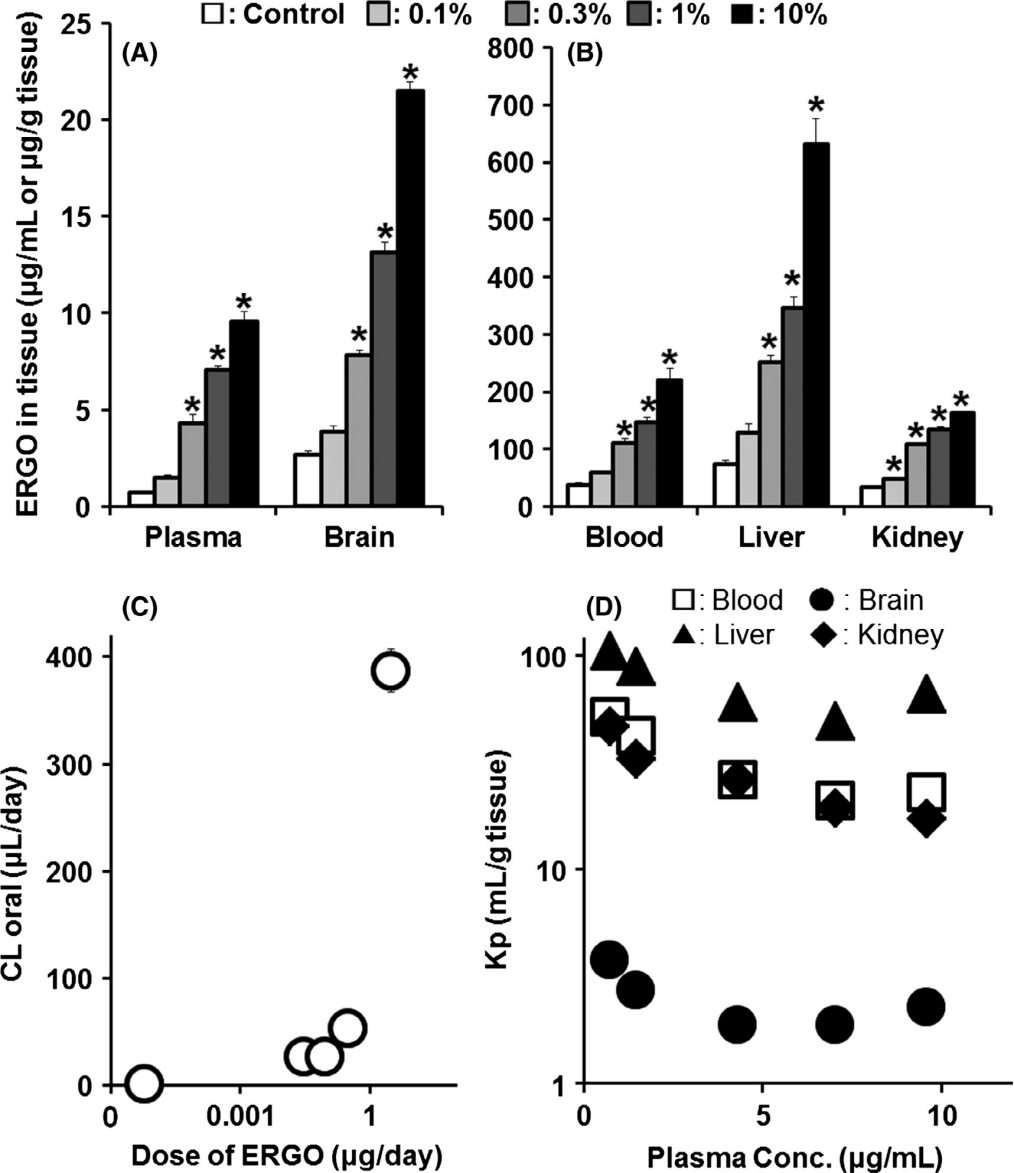
ERGO (Ergothioneine) concentrations in the body and pharmacokinetic parameters for ERGO in mice fed diet containing GOME. (A) ERGO concentrations in plasma and brain, and (B) those in blood, liver, and kidney were measured by LC‐MS/MS in mice given normal diet (white columns), or diet containing 0.1% (light gray columns), 0.3% (gray columns), 1% (dark gray columns), or 10% (black columns) GOME for 2 weeks. (C) CL_oral_ of ERGO was calculated from plasma concentration and daily intake of ERGO. (D) Kp in blood (open squares), brain (closed circles), liver (closed triangles), and kidney (closed diamonds) was calculated from the ERGO concentrations in plasma and each tissue. Each point and vertical bar represents the mean ± SEM. (*n* = 4). *P <* *0.05, significant difference from the corresponding control value.

First, we examined the distribution of ERGO to the brain parenchyma across the BBB (blood–brain barrier). We measured the concentration of ERGO in blood, plasma, brain, liver, and kidney at 2 weeks after the mice had been started on diet containing 0.1–10% GOME (w/w; Fig. 1A). ERGO concentration in each tissue was dose‐dependently increased [Plasma *F*(4,15) = 133.9, *P *<* *0.01; Brain *F*(4,15) = 447.8, *P *<* *0.01; Blood *F*(4,15) = 47.0, *P *<* *0.01; Liver *F*(4,15) = 87.3, *P *<* *0.01; Kidney *F*(4,15) = 298.9, *P *<* *0.01] (Fig. 2A and B). ERGO concentration in the brain was similar to that in plasma at every dosage (Fig. 2A). ERGO concentrations in the brain and liver of the group given diet containing 10% GOME were at most 1.5 times higher than those in animals given diet containing 1% GOME (w/w), indicating nonlinear behavior. A similar tendency was seen in the case of plasma, blood, and kidney, although the differences in ERGO concentration between the two doses were smaller (Fig. 2A and B). To directly evaluate saturation of ERGO disposition, CL_oral_ and Kp of ERGO were calculated. As the dosage of GOME was increased, CL_oral_ increased (Fig. 2C), while Kp decreased (Fig. 2D). The decrease in Kp occurred at the plasma ERGO concentration of about 1–2 *μ*g/mL (4–8 *μ*mol/L), which is close to the Michaelis constant (Km) for ERGO of OCTN1 (~5 *μ*mol/L, Kato et al. 2010). These results indicate that ERGO is highly distributed to the brain after oral ingestion.

### Antidepressant‐like effect of ERGO

**Figure 3:**
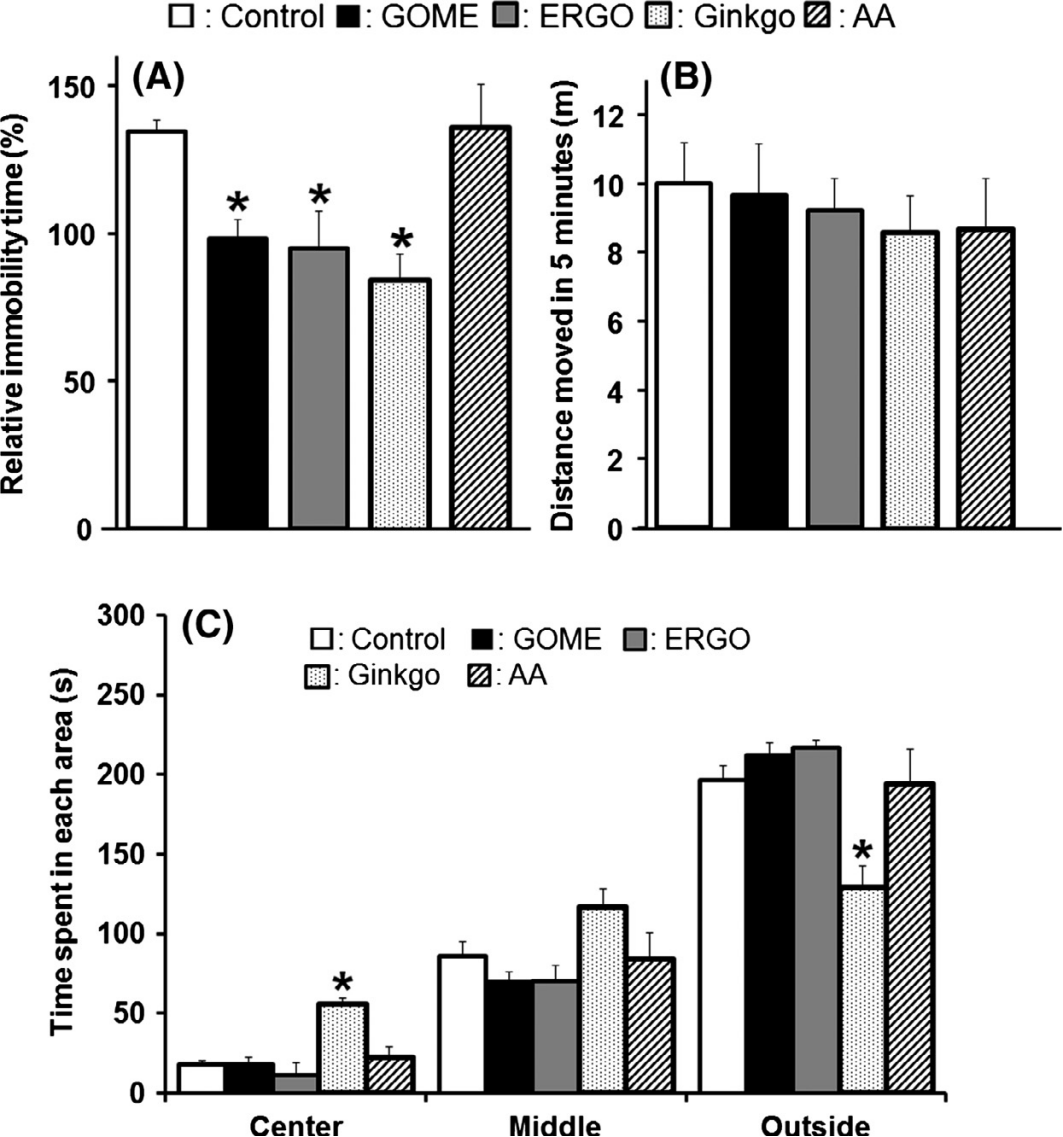
Behavioral test in mice given diet containing GOME, ERGO (ergothioneine), GBE (*Ginkgo biloba* extract), or AA. Mice were given normal diet (white columns), or diet containing 10% GOME (black columns), authentic ERGO (dark gray columns), GBE (dotted columns), or AA (diagonal columns) for 2 weeks. (A) Immobility time in 5 min of FST was measured in mice. Increase in immobility time was calculated from the values after each diet treatment and before the start of the treatment. Each value is the mean ± SEM. (Control: *n* = 11, GOME:* n* = 11, ERGO:* n* = 6, GBE:* n* = 6, AA:* n* = 6). (B) Locomotor activity of mice. Each mouse was placed in an individual cage, and locomotion was assessed for 5 min after habituation. Each value is shown as mean ± SEM. (*n* = 6). (C) Time spent in the center, middle, and outside zones was measured. Each mouse was placed in a novel chamber for 5 min. Each value is the mean ± SEM. (Control: *n* = 15, GOME:* n* = 15, ERGO:* n* = 6, GBE:* n* = 3, AA:* n* = 6). *P <* *0.05, significant difference from the corresponding control value.

**Figure 4:**
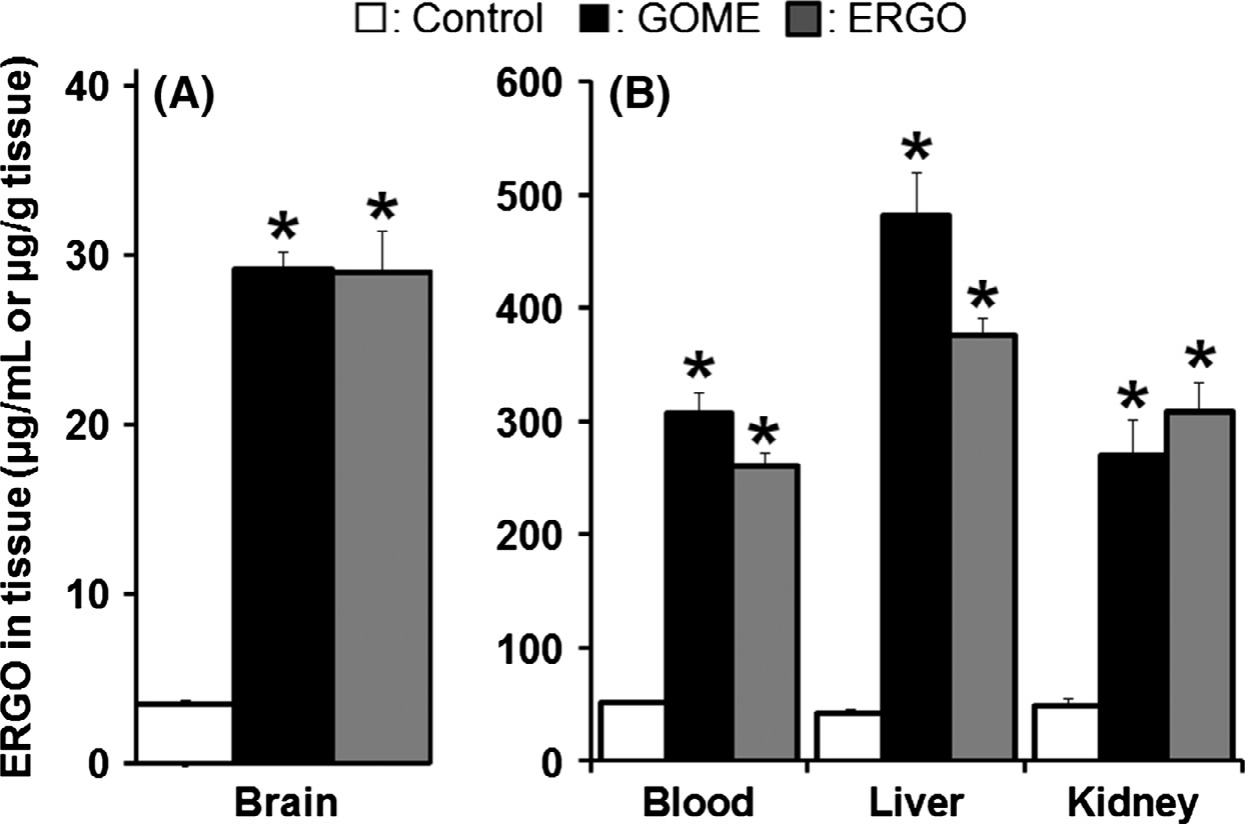
Comparison of ERGO (ergothioneine) concentrations in the body after oral ingestion of GOME or ERGO. (A) ERGO concentration in brain, and (B) those in blood, liver, and kidney were measured by HPLC in mice given normal diet (white columns), or diet containing 10% GOME (black columns), or authentic ERGO (dark gray columns) for 2 weeks. Each vertical bar represents the mean ± SEM. (*n* = 6). *P <* *0.05, significant difference from the corresponding control value.

Next, we examined the antidepressant‐like effect of ERGO. GOME or ERGO was fed to mice in the diet for 2 weeks, and FST, which is a well‐established tool for assessing antidepressant‐like activity in rodents, was performed (Fig. 1B). The immobility time of mice given diet containing 10% GOME was significantly lower than that of mice given normal diet [*F*(5,70) = 2.9, *P *<* *0.05] (Fig. 3A). To examine whether this activity of GOME is due to ERGO present in GOME, mice were fed diet containing authentic ERGO, the amount of which (120 mg ERGO/100 g diet) was equivalent to that in diet containing 10% GOME. The immobility time in mice given diet containing 10% GOME was similar to that given diet containing the equivalent amount of authentic ERGO (Fig. 3A). In addition, ERGO concentration in the brain in mice given diet containing 10% GOME was also similar to that given diet containing the equivalent amount of authentic ERGO (Fig. 4). These data suggested that the antidepressant‐like effect of GOME is predominantly due to ERGO contained in the extract.

*Ginkgo biloba* extract, which is known to have an antidepressant‐like effect in rodents (Sakakibara et al. 2006; Rojas et al. 2011) and was used as a positive control in this study, also decreased the immobility time, whereas AA, a hydrophilic antioxidant like ERGO, had no effect on the immobility time (Fig. 3A). To rule out the possibility that the decrease in the immobility time was due to an increase in motor activity, a locomotor activity test was performed. We found no difference in motor activity among the groups given control diet and diets containing GOME, ERGO, GBE, or AA for 2 weeks (Fig. 3B), suggesting that differences in motor activity cannot account for the differences in immobility time observed in this study.

To examine the antianxiety effect of ERGO in addition to the antidepressant‐like effect, we employed an open field test. The residence time of mice in each zone showed no significant difference between the control group and the groups given diet containing GOME, ERGO or AA (Fig. 3C). On the other hand, residence time in the center zone after ingestion of GBE was significantly longer than that of the control group [*F*(4,40) = 7.4, *P *<* *0.01] (Fig. 3C). A similar tendency was observed in the middle zone (Fig. 3C). Concomitantly, residence time in the outer zone was significantly shorter in the GBE ingestion group than the control group [*F*(4,40) = 4.9, *P *<* *0.01] (Fig. 3C). These results suggest that GBE may have an antianxiety effect, but ERGO does not.

**Figure 5:**
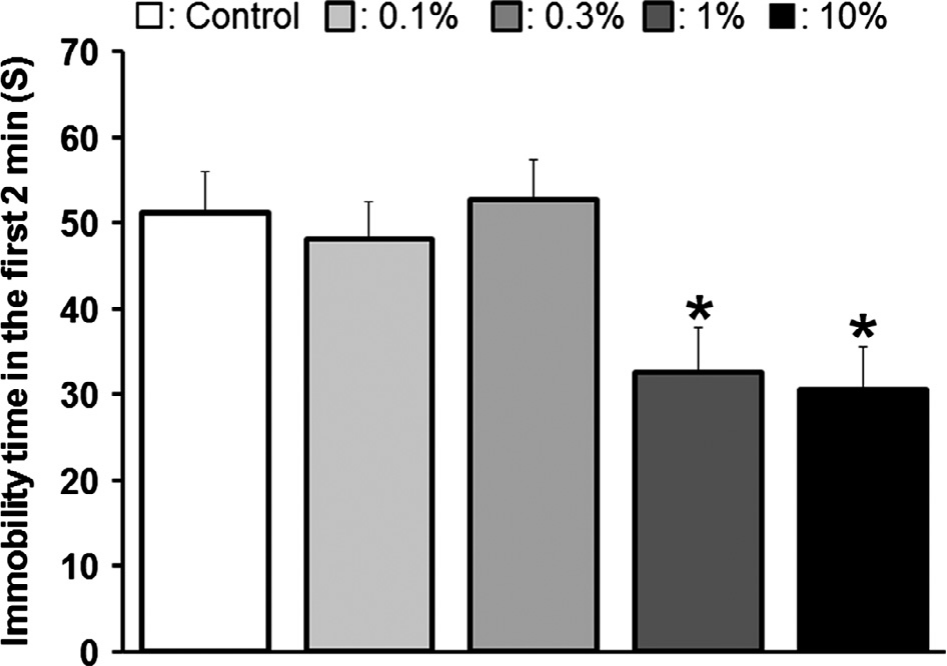
Dose‐dependent reduction in immobility time in TST after oral ingestion of GOME. Mice were given normal diet (white column, *n* = 15), or diet containing 0.1% (light gray column, *n* = 6), 0.3% (gray column, *n* = 6), 1% (dark gray column, *n* = 12), or 10% (black column, *n* = 15) GOME for 2 weeks and subjected to TST. Immobility time in 2 min was measured. Each value is the mean ± SEM. *P <* *0.05, significant difference from the corresponding control value.

To confirm the antidepressant‐like effect of ERGO and examine its dose dependency, TST (tail suspension test) was also performed (Fig. 1A). The immobility time of mice given diet containing 1% or 10% GOME was decreased compared to that of mice given normal diet [*F*(4,49) = 3.8, *P *<* *0.01] (Fig. 5), supporting the idea that ERGO possesses an antidepressant‐like effect. These results suggested that ingestion of 1% or more of GOME, namely 12 mg/100 g diet of ERGO or more, for 2 weeks has an antidepressant‐like effect.

### Promotion of neuronal differentiation by ERGO

**Figure 6:**
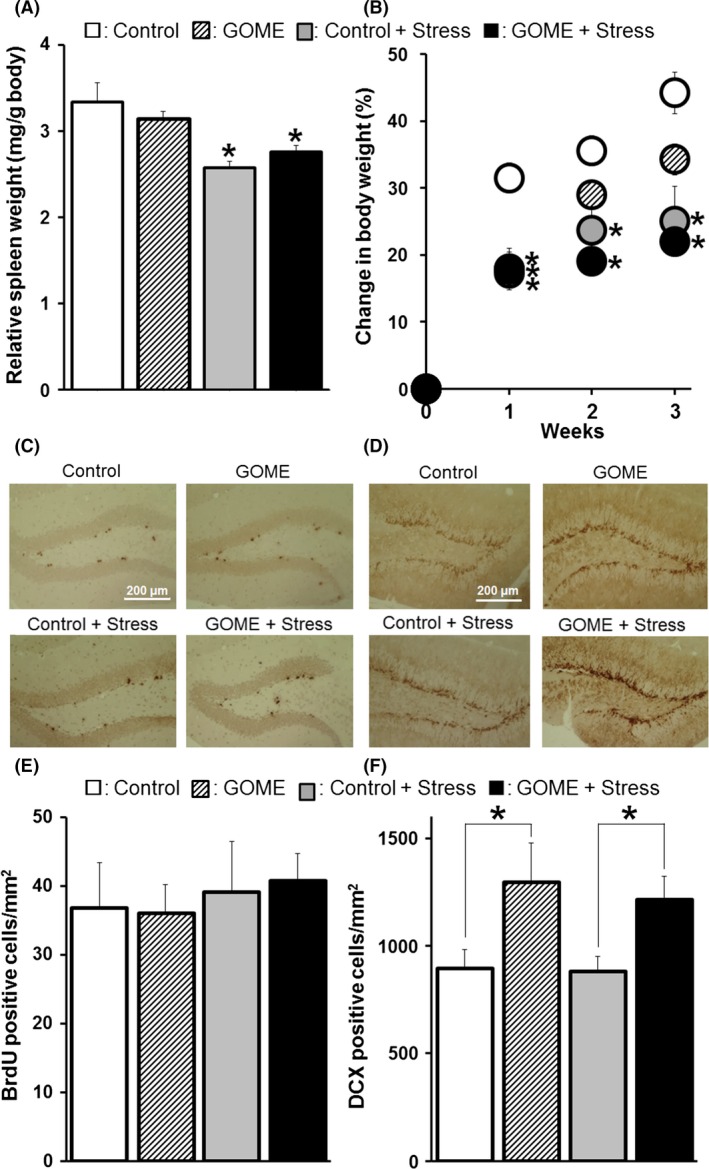
Bromodeoxyuridine incorporation or DCX expression in mice given diet containing GOME under a chronic stress condition. Mice were given normal diet ((A), (B) white symbols, (C), (D) upper left panels) or diet containing 10% GOME ((A), (B) diagonal symbols, (C), (D) upper right panels) under a normal condition, or normal diet ((A), (B) gray symbols, (C), (D) lower left panels), or diet containing 10% GOME ((A), (B) black symbols, (C), (D) lower right panels) under a chronic stress condition, for 21 days. Stress was imposed by forcing the animals into an immobilizer for 4 h/day. (A) Spleen weight was measured and normalized by body weight. Each value is shown as the mean ± SEM. (*n* = 3). (B) Change in body weight of each mouse was measured every week and normalized with that at the start of restraint stress. Each value is shown as the mean ± SEM. (*n* = 5). Typical phase‐contrast micrographs of (C) BrdU‐incorporating cells and (D) DCX‐expressing cells in each group are shown. The numbers of (E) BrdU‐incorporating cells and (F) DCX‐expressing cells were counted by using a cell image analyzer in at least two areas from four to eight individual mice in each group. Vertical bar represents the mean ± SEM. (Control: *n* = 8, GOME:* n* = 8, Control + Stress: *n* = 15, GOME + Stress: *n* = 14). *P <* *0.05, significant difference from the corresponding control value.

Some antidepressant drugs promote neurogenesis, and this activity may be related to their antidepressant effect. So, we next examined promotion of neurogenesis by ERGO. It has been reported that stress decreases neurogenesis in the hippocampus (Kim et al. 2011). Thus, promotion of neurogenesis by ERGO may be more clearly observed under a stress condition rather than a normal one. In addition, since we expected to examine whether ERGO could reverse depression‐like symptoms by stress, the repeated restraint stress procedure by Kim et al. (2011) was employed in this study. The immobility time of the stressed mice was significantly longer than that of the nonstressed mice in FST and TST (Kim et al. 2011). Therefore, 10% GOME was fed to mice for 3 weeks under either a normal or a stress condition (Fig. 1C). Spleen weight [*F*(3,8) = 7.6, *P *<* *0.05] and body weight [First week *F*(3,16) = 7.8, *P *<* *0.01; Second week *F*(3,16) = 8.8, *P *<* *0.01; Third week *F*(3,16) = 8.9, *P *<* *0.01] in the stress group was significantly less than in the control group (Fig. 6A and B), confirming the expected effect of stress loading. However, behavioral changes were not observed between two groups in TST (data not shown). Administration of GOME and/or stress loading did not affect the number of cells positive for BrdU, a proliferating cell marker (Fig. 6C and E). The number of cells positive for DCX, an immature neuronal marker, was increased in the GOME group compared to the control group, regardless of stress [*F*(3,41) = 3.7, *P *<* *0.05] (Fig. 6D and F), suggesting that ERGO may promote neuronal differentiation in vivo.

## Discussion

The present findings indicate that the food‐derived antioxidant ERGO is highly distributed to the brain across the BBB after oral ingestion and exert the antidepressant‐like effect despite its hydrophilic property. ERGO has been detected in the brain of mice under usual dietary conditions (Kato et al. 2010), but there has been no direct evidence that ERGO can permeate the BBB and be distributed to the brain. In this study, we clarified the relationship between oral intake and brain parenchymal content of ERGO (Fig. 2). The observed increase in CL_oral_ (Fig. 2C) and decrease in Kp in various organs including the brain (Fig. 2D) with increasing level of ERGO in the diet suggest saturation in the disposition kinetics of ERGO in the body. In particular, since the decrease in Kp was observed at the plasma concentration close to the Km (5 *μ*mol/L) of ERGO for OCTN1 (Fig. 2D), it seems likely that the distribution of ERGO to each organ is mainly governed by OCTN1. However, OCTN1 is minimally expressed in the BBB (Okura et al. 2008). Therefore, ERGO may be delivered to the brain parenchyma by transporters other than OCTN1. OCTN1 is, on the other hand, functionally expressed in brain neurons (Nakamichi et al. 2012), and therefore, the saturation of brain distribution of ERGO (Fig. 2D) may reflect saturation of uptake into neurons after passage through the BBB.

The oral ingestion of diet containing 10% GOME and the same amount of authentic ERGO as it had similar effects on immobility time of mice in FST (Fig. 3A), and the concentration of ERGO in the brain was also similar in mice given the two diets (Fig. 4A), suggesting that the antidepressant‐like effect of GOME may be predominantly due to ERGO. ERGO shows low membrane permeability due to its high water‐solubility, but is a good substrate of OCTN1. We have previously reported that disposition of food‐derived ERGO under normal dietary conditions is mainly regulated by OCTN1, since ERGO is present in various organs of wild‐type mice, but absent in those of *octn1* gene knockout mice (Kato et al. 2010). In this study, we found that even when a higher amount of ERGO was loaded into mice by ingestion of GOME, the ERGO disposition was still governed by OCTN1, showing nonlinear kinetics in plasma concentration and tissue distribution (Fig. 2). This result is consistent with the idea that disposition of ERGO is controlled by the transporter. Such transporter‐mediated distribution of ERGO to the body is clearly distinct from the case of flavonoids, major components of GBE exerting various biological activities, which do not have specific uptake transporters in the body. On the other hand, ingestion of AA, which is a water‐soluble antioxidant like ERGO, did not decrease the immobility time of mice in FST (Fig. 3A). AA minimally permeates the BBB and is distributed to the brain only in the form of oxidized dehydroascorbic acid (Gess et al. 2011). Moreover, AA did not affect neuronal differentiation, whereas ERGO promoted it in cultured neural stem cells (Ishimoto et al. 2014). These may be the reason why oral ingestion of AA failed to show any antidepressant‐like effect.

*Ginkgo biloba* extract also decreased the immobility time of mice as effectively as ERGO in FST (Fig. 3A). It has already been reported that GBE exhibits its antidepressant‐like effect via modulation of serotonergic and dopaminergic neurotransmission, or antioxidant enzymes in rodents (Sakakibara et al. 2006; Rojas et al. 2011). Spontaneous locomotor activity showed no difference among the groups given GOME, ERGO, and GBE in this study (Fig. 3B), indicating that the decrease in immobility time after ingestion of these materials is not due to an increase in motor activity. On the other hand, in the open field test to evaluate anxiety, GBE increased the residence time in the center zone and decreased that in the outer zone compared to the control group, as reported previously (Ma et al. 2012), whereas GOME and ERGO had no such effect (Fig. 3C). Mice usually prefer to stay on the wall side (outer zone) in a novel environment (Crawley 1999), and administration of drugs with antianxiety and/or excitatory effects, such as cocaine, decreases the residence time in the outer zone (Prut and Belzung 2003; Carey et al. 2008). Thus, since ERGO shows the different pattern of neurological effect from GBE, and further studies are required to clarify whether ERGO exerts the antidepressant‐like effect via a different mechanism from GBE.

The concentration of ERGO in the brain was increased dose‐dependently after oral ingestion of GOME [13.1 *μ*g/mL (57 *μ*mol/L) in the group given 1% GOME diet and 21.5 *μ*g/mL (94 *μ*mol/L) in the group given 10% GOME diet (Fig. 2A)], suggesting that 57 *μ*mol/L ERGO is a sufficient concentration to exert an antidepressant‐like effect. On the other hand, the concentration of ERGO in blood of mice given control diet was 37 *μ*g/mL (161 *μ*mol/L), which is similar to the blood concentration in healthy volunteers, 40 *μ*g/mL (174 *μ*mol/L) (Kato et al. 2010). In addition, GOME used in this study contains approximately 1.2% ERGO and mice (20 g body weight) took 3.68 g of diet per day (Kato et al. 2009), so that daily intake of ERGO was about 22 mg/kg body weight in mice given diet containing 1% GOME. The body surface area per weight of human is about 15.4 times smaller than that of mice (Davies and Morris 1993), so ERGO may exert an antidepressant effect in human at 1/15.4 of the concentration in mice. For a 70 kg human, the required intake of ERGO per day would be 22 × 70/15.4 = 100 mg (about 8 g GOME), and this seems to be an amount that could be easily ingested. Thus, it is plausible that an antidepressant effect in humans could be achieved by taking moderate amounts of GOME.

This study is the first to indicate that ERGO‐containing diet GOME promotes neuronal differentiation in the hippocampus dentate gyrus in vivo (Fig. 6), and this result is compatible with the previous report that exposure of cultured neural stem cells to ERGO markedly increased the number of cells immunoreactive for the neuronal marker *β*III‐tubulin, suggesting that ERGO promotes neuronal differentiation in vitro (Ishimoto et al. 2014). In this study, whereas an ERGO‐containing diet increased DCX‐positive neuronal cells (Fig. 6E and F), the diet did not affect the number of BrdU‐positive proliferating cells (Fig. 6C and D). ERGO suppresses proliferation of neural stem cells by reducing cellular oxidative stress at least in vitro since exposure of cultured neural stem cells to ERGO or other antioxidants (edaravone and AA) led to a significant decrease in the area of neurospheres, which are formed from clusters of proliferating cells, with concomitant elimination of intracellular reactive oxygen species (Ishimoto et al. 2014). Such two different effects of ERGO, suppression of proliferation and promotion of neuronal differentiation, on neural stem cells (Ishimoto et al. 2014) could be one of the reasons for the discrepancy of the number between BrdU‐ and DCX‐positive cells (Fig. 6). Several antidepressant drugs such as fluoxetine, imipramine, and reboxetine, promote neurogenesis (Malberg et al. 2000; Santarelli et al. 2003; Ohira et al. 2013). Thus, ERGO may exert its antidepressant‐like effect at least partially via promotion of neuronal differentiation in vivo. However, further studies would be required to confirm this.

In conclusion, orally ingested ERGO is transported across the BBB into the brain and exerts the antidepressant‐like effect in mice. Since ERGO is abundantly contained in edible golden oyster mushrooms, and its disposition is limited by the uptake transporter OCTN1, it is likely to be safe for long‐term use with few or no side effects in human. Furthermore, ERGO could exert an antidepressant effect at plausibly achieved level of daily ingestion according to the estimate from the present results. Thus, we suggest that ERGO is a promising candidate for prevention and/or treatment of depression. In the future, however, we need to show that oral ingestion of ERGO or its derivatives prevents onset of depression in stressed people and alleviates their symptoms in depressed patients.

## Conflict of Interest

There is no conflict of interest statement.
